# Vascular calcification on the risk of kidney stone: a meta-analysis

**DOI:** 10.1080/0886022X.2023.2183727

**Published:** 2023-03-03

**Authors:** Linxi Huang, Junjie Hu, Cheng Xue, Jiarong Ding, Zhiyong Guo, Bing Yu

**Affiliations:** a Department of Nephrology, Changhai Hospital, Naval Medical University (Second Military Medical University), Shanghai, China; b Department of Cell Biology, Center for Stem Cell and Medicine, Naval Medical University (Second Military Medical University), Shanghai, China; c Division of Nephrology, Kidney Institute of CPLA, Changzheng Hospital, Naval Medical University (Second Military Medical University), Shanghai, China

**Keywords:** Kidney stone, vascular calcification, risk assessment, meta-analysis

## Abstract

**Background:**

The association between vascular calcification (VC) and kidney stone is still inconclusive. Therefore, we conducted a meta-analysis to estimate the risk of kidney stone disease in subjects with VC.

**Methods:**

To identify publications from related clinical studies, we performed a search on PubMed, Web of Science, Embase, and Cochrane Library databases from their inceptions until 1 September 2022. According to obvious heterogeneity, a random-effects model was used to calculate the odds ratios (ORs) and corresponding 95% confidence intervals (CIs). Subgroup analysis was conducted trying to dissect the effects of VC in different segments and population regions in predicting kidney stone risk.

**Results:**

Seven articles were included with a total number of 69,135 patients, of which 10,052 have vascular calcifications and 4728 have kidney stones. There was a significantly higher risk of kidney stone disease in participants with VC versus control (OR = 1.54, 95% CI: 1.13–2.10). Sensitivity analysis confirmed the stability of the results. VC can be separated into abdominal, coronary, carotid, and splenic aortic calcification while pooled analysis of abdominal aorta calcification did not indicate a significant higher kidney stone risk. An obvious higher risk of kidney stone was observed in Asian VC patients (OR = 1.68, 95% CI: 1.07–2.61).

**Conclusion:**

Combined evidence of observational studies suggested patients with VC may be associated with an increased risk of kidney stone disease. Despite the predictive value was relatively low, it is still worth noting that patients with VC are under the threat of kidney stone disease.

## Introduction

Kidney stone disease is a global health problem affecting approximately 10% adult population over the world [1]. In the past decades, the prevalence of kidney stone increased worldwide from 3.2% to 8.8% in American population, as well as in European and Asian population reaching current prevalence of 5–10% in Europe and 1–19% in Asia [2–4]. Notably, kidney stone disease has become an increasing contributor to chronic kidney disease (CKD), including end stage renal disease [5,6]. Kidney stone itself as well as accompanied CKD is of confirmed association with higher risk of all-cause mortality [7,8]. In that case, early intervention to kidney stone is highly promising to reduce health burden.

Moreover, observational studies have indicated associations between myocardial infarction or coronary heart disease (CHD) and stroke in kidney stone formers [9–11]. Coronary artery calcium is a specific marker of coronary atherosclerosis, the most common cause of CHD [12]. Extra-coronary calcification, take thoracic and abdominal aortic calcification for example, is also implied as a strong predictor of future cardiovascular events [13,14]. Vascular calcification is a pathologic result of aberrant calcium and phosphate levels, which is marked with precipitation of calcium phosphate in arterial vessels [15]. Nevertheless, the most common stone components are calcium-based, with oxalate or phosphate, which represented over 75% of all stone phenotypes [16]. Several systematic comorbidities, including obesity, diabetes mellitus, and hypertension, are reported to be factors contributing to both kidney stone and vascular calcification [17,18]. More researches are evolving focusing on the links between vascular calcification and kidney stone. Calcium sensing receptor, expressed in calcitropic tissues including kidney and vascular system, is responsible for calcium homeostasis and associated with formation of nephrolithiasis and vascular calcification [19]. Bone forming or ossification mechanism is also involved in early stage of kidney stone formation and arterial calcification [20]. Other abnormities including endothelial dysfuction and pyrophosphate deficiency are also reported common promotors [21,22]. The shared pathogenesis strongly indicated a predict value of vascular calcification on kidney stone disease with early interventions to avoid advanced CKD, CHD and death.

Several observational studies have reported associations between kidney stone occurrence and coronary artery calcium score, carotid atherosclerosis or increased systemic calcification in abdominal and splenic aortic vessels [23–25]. However, inconsistent research conclusions also emerged. Daniel Schoenfeld et al. reported that the presence of abdominal aortic calcification (AAC) or AAC severity score was not associated with the risk of kidney stone [26]. At present, no study has comprehensively assessed the risk of vascular calcification on kidney stone disease despite the epidemiological and pathologic links [21]. Therefore, we conducted a meta-analysis to illustrate the kidney stone risk burden in patients with vascular calcification. In that case, nephrologists should be more alert to kidney stone injury when vascular calcification is observed.

## Materials and methods

This meta-analysis was conducted and reported according to the Preferred Reporting Items for Systematic Reviews and Meta-Analyses statement (PRISMA) [27] and registered in PROSPERO (https://www.crd.york.ac.uk/PROSPERO/) as CRD42022357772. The report details are presented in Supplementary table 1.

### Literature search and selection strategy

**Figure 1.**
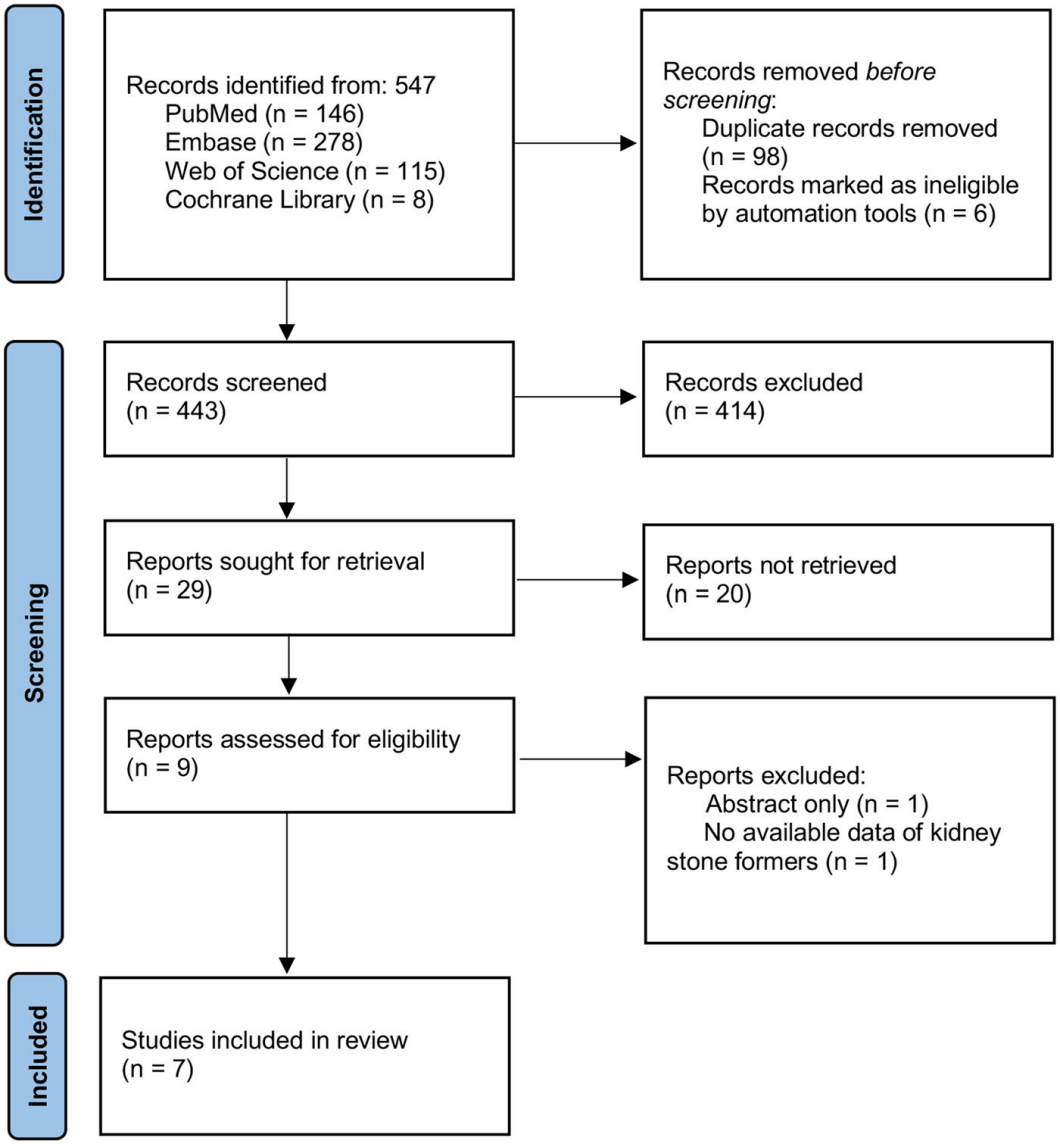
Flow chart of the literature search and study selection.

We conducted a systematic review of the published literatures in electronic databases PubMed, Embase, Web of Science and Cochrane Library from their inceptions until 1 September 2022. The details of search strategies and results in mentioned databases are presented in Supplementary table 2. No restrictions to languages, regions and publication types were set. Two reviewers (Huang Linxi and Hu Junjie) independently screened article titles, abstracts and full texts to identify potential studies. The inclusion criteria were followed: (1) observational or cohort studies were eligible; (2) subjects were diagnosed with kidney stone or vascular calcification. Reviews, case reports, comments and duplicates were removed. The main flow-work was diagramed in Figure 1, and any disagreement in the procedure was resolved by discussion.

### Data extraction and quality assessment

Two independent reviewers (Huang Linxi and Xue Cheng) collected data from identified correlative studies and assessed the risks of bias. Any conflicting evaluations were solved by negotiations or consulting the third author (Guo Zhiyong). Information extracted from these studies included name of the first author, publication year, study district, study design, sample size and diagnostic evidence. We also assessed the risk of bias of included studies according to the basis of the Newcastle-Ottawa scale [28,29]. Table 1 presented the score of each study.

**Table 1.** Assessment of the quality of the studies according to the NOS.

Study	Year	Selection				Comparability	Outcome assessment			Quality score
		①	②	③	④	⑤	⑥	⑦	⑧	
Shavit	2015	Yes	Yes	Yes	Yes	Yes	Yes	Yes	No	7
Patil	2016	Yes	Yes	Yes	Yes	Yes	Yes	Yes	No	7
Tanaka	2017	Yes	Yes	Yes	Yes	Yes	Yes	Yes	No	7
Kim	2018	Yes	Yes	Yes	Yes	Yes	Yes	Yes	No	7
Stern	2019	Yes	Yes	Yes	Yes	Yes	Yes	Yes	Yes	8
Schoenfeld	2021	Yes	Yes	Yes	Yes	Yes	Yes	Yes	No	7
Li	2022	Yes	Yes	Yes	Yes	Yes	Yes	Yes	No	7

① Representativeness of the exposed cohort; ② selection of the non-exposed cohort; ③ ascertainment of exposure; ④ demonstration that outcome of interest was not present at start of study; ⑤ comparability of cohorts on the basis of the design or analysis; ⑥ assessment of outcome; ⑦ was follow-up long enough for outcomes to occur; ⑧ adequacy of follow up of cohorts.

### Data synthesis and statistical analyses

All statistical analyses were performed using Review Manager 5.3 (Cochrane Collaboration, Oxford, UK) and R (version 4.0.3). The odds ratios (ORs) were used to compare dichotomous variables, and entire results were reported with 95% confidence intervals (CIs). Statistical heterogeneities among articles were assessed using the chi-square test, and heterogeneity was calculated using the *I^2^* statistic. *I^2^* ranges of 0–25%, 25–75% and over 75% are regarded as low, moderate and considerable heterogeneity, respectively. The random-effects model was used because of considerable heterogeneity between articles. Sensitivity analysis was conducted by excluding each enrolled study. Funnel plot, Egger and Begg tests were used to evaluate publication bias [30].

## Results

### Literature collection and characteristics

From the literature search in electronic databases, a total of 547 publications were retrieved. After duplicates removal, 98 records were excluded. six records identified from Cochrane Library were registered clinical trials and were ineligible. Screening of titles and abstracts excluded 414 records. Then the full texts of remaining 29 articles were retrieved for further evaluation. Of these, reviews, case reports, comments and animal researches were excluded and two clinical studies did not provide available numbers of patients diagnosed with kidney stone or vascular calcification. Thus, seven studies involving 69,135 participants were ultimately included for quantitative synthesis (Figure 1). The majority of the included studies, 6 out of 7, were case–control studies. Mere one study was cross-sectional study [31]. Among the enrolled participants, 10,052 patients were diagnosed with vascular calcification, and 4,728 patients with urolithiasis. Most studies, 5 out of 7, focused on abdominal aortic calcification, while Stern et al. reported splenic artery calcification simultaneously [25]. The other two studies reported carotid and coronary artery calcifications respectively [31,32]. Six studies utilized computed tomography (CT) imaging as evidence for aortic calcification. Li et al. also referred to abdominal vascular ultrasound to evaluate vascular calcification [33]. Carotid artery calcification was examined by panoramic radiographs [32]. Coronary artery calcification was assessed by cardiac tomography [31]. The diagnosis of kidney stones was also based on imaging evidence, including CT scans, X-ray photography and ultrasound examinations. The other main characteristics of the studies are summarized in Table 2. In accordance with the Newcastle–Ottawa quality assessment for retrospective studies, we assessed the possible bias of each included study (Table 1). All included studies achieved at least 7 scores, indicating good quality.

**Table 2.** Characteristics of the enrolled studies.

Study	Publication year	Study design	District	Center	Sample size (male)	Age (year)	Body mass index (kg/m^2^)	Vascular calcification evaluation	Type of vascular calcification	Kidney stone evaluation
Shavit [34]	2015	Case-control study	UK	1	111 (63)	47 ± 13 for controls, 47 ± 14 for KSFs	NA	CT scans	Abdominal aortic calcification	Non-contrast CT scans of the abdomen and the pelvis
Patil [32]	2016	Case-control study	Saudi Arabia	1	240 (144)	41.1 ± 6.7 for controls, 40.6 ± 7.8 for KSFs	27.1 ± 4.6 for controls, 28.6 ± 4.8 for KSFs	Panoramic radiographs	Carotid artery calcification	Radiographic findings
Tanaka [35]	2017	Case-control study	Japan	2	439 (258)	62 (54–72) for controls, 63 (54–72) for KSFs	24 ± 4 for controls, 25 ± 4 for KSFs	Abdominal CT	Abdominal aortic calcification	Abdominal CT
Kim [31]	2018	Cross-sectional study	South Korea	3	62,091 (48,369)	41.5 ± 7.6 for all participants	24.3 ± 3.2 for all participants	Cardiac tomography	Coronary artery calcification	Ultrasonography of the abdomen
Stern [25]	2019	Case-control study	America	1	394 (156)	43 (19-67) for controls, 43 (19-75) for KSFs	27.5 for controls, 27.6 for KSFs	Non-contrast CT scans	Abdominal aortic and splenic artery calcification	Non-contrast CT scans
Schoenfeld [26]	2021	Case-control study	America	1	1344 (537)	51.9 ± 15.4 for controls, 51.8 ± 15.5 for KSFs	29.8 ± 7.2 for controls, 29.7 ± 6.9 for KSFs	Non-contrast CT scans	Abdominal aortic calcification	Non-contrast abdominal CT scans
Li [33]	2022	Case-control study	China	1	4516 (2749)	49.0 ± 11 for all participants	24.2 ± 3.3 for all participants	CT scans, abdominal vascular ultrasound	Abdominal aortic calcification	Abdominal CT (contrast and non-contrast), KUB, urinary ultrasound

Abbreviations: KSF, kidney stone formers; CT, computed tomography; KUB, kidney–ureter–bladder X-ray photography; NA, not available.

### Results of vascular calcification versus control

**Figure 2.**
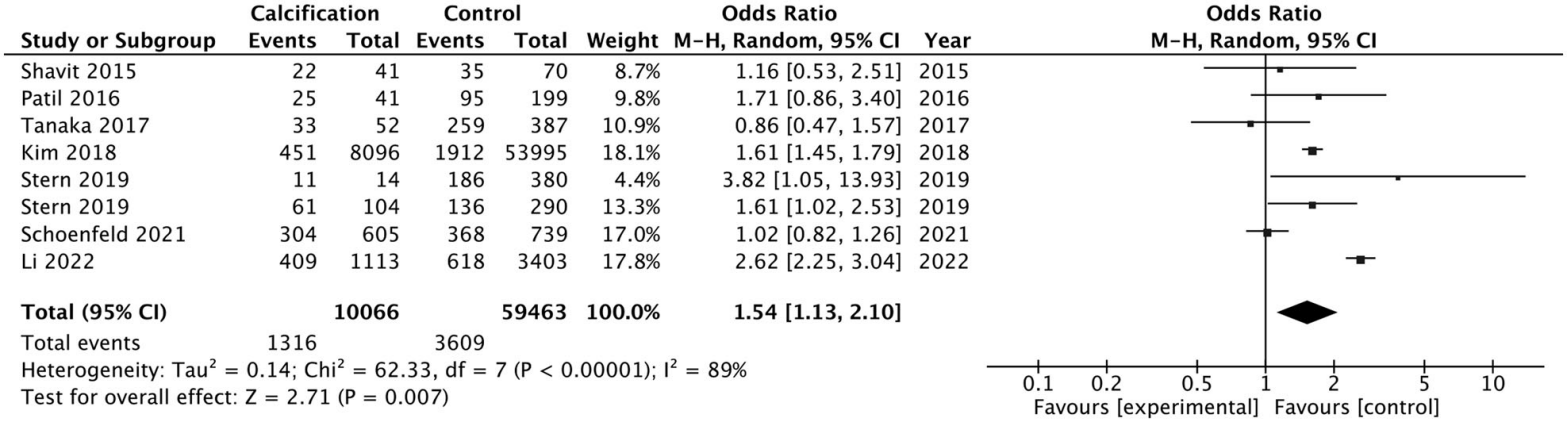
Pooled odds ratio of kidney stone disease in patients with vascular calcification compared with healthy control.

**Figure 3.**
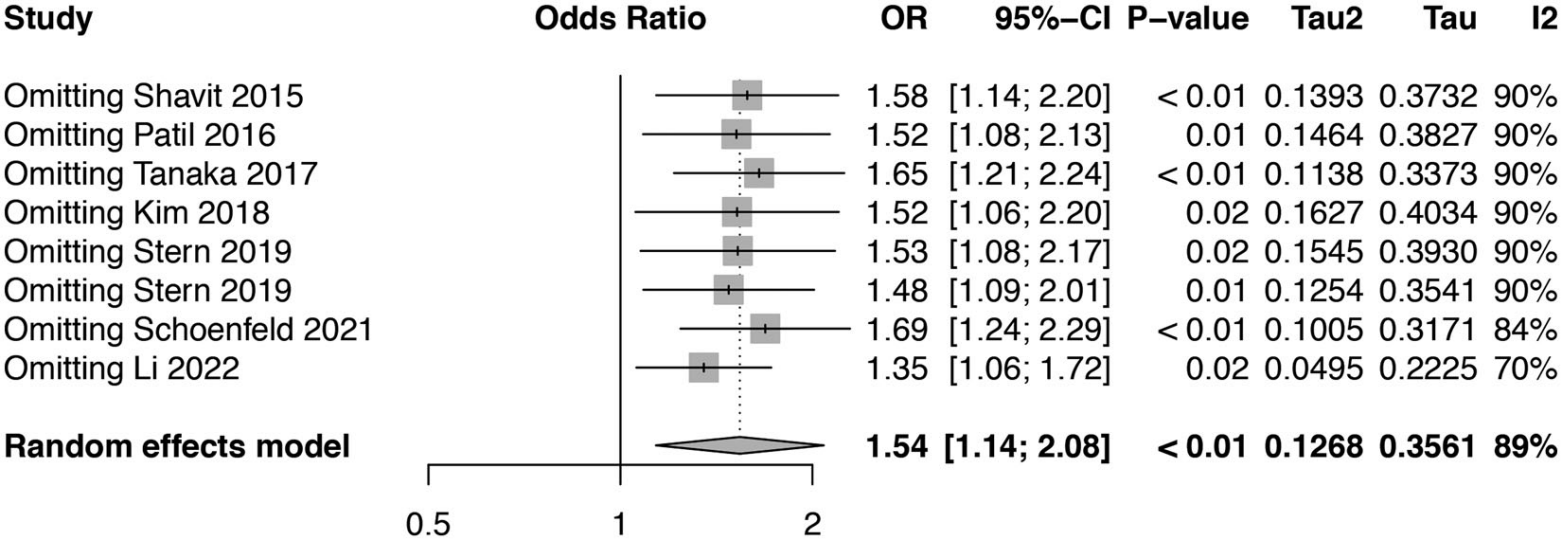
Forest plot of sensitivey analysis.

**Figure 4.**
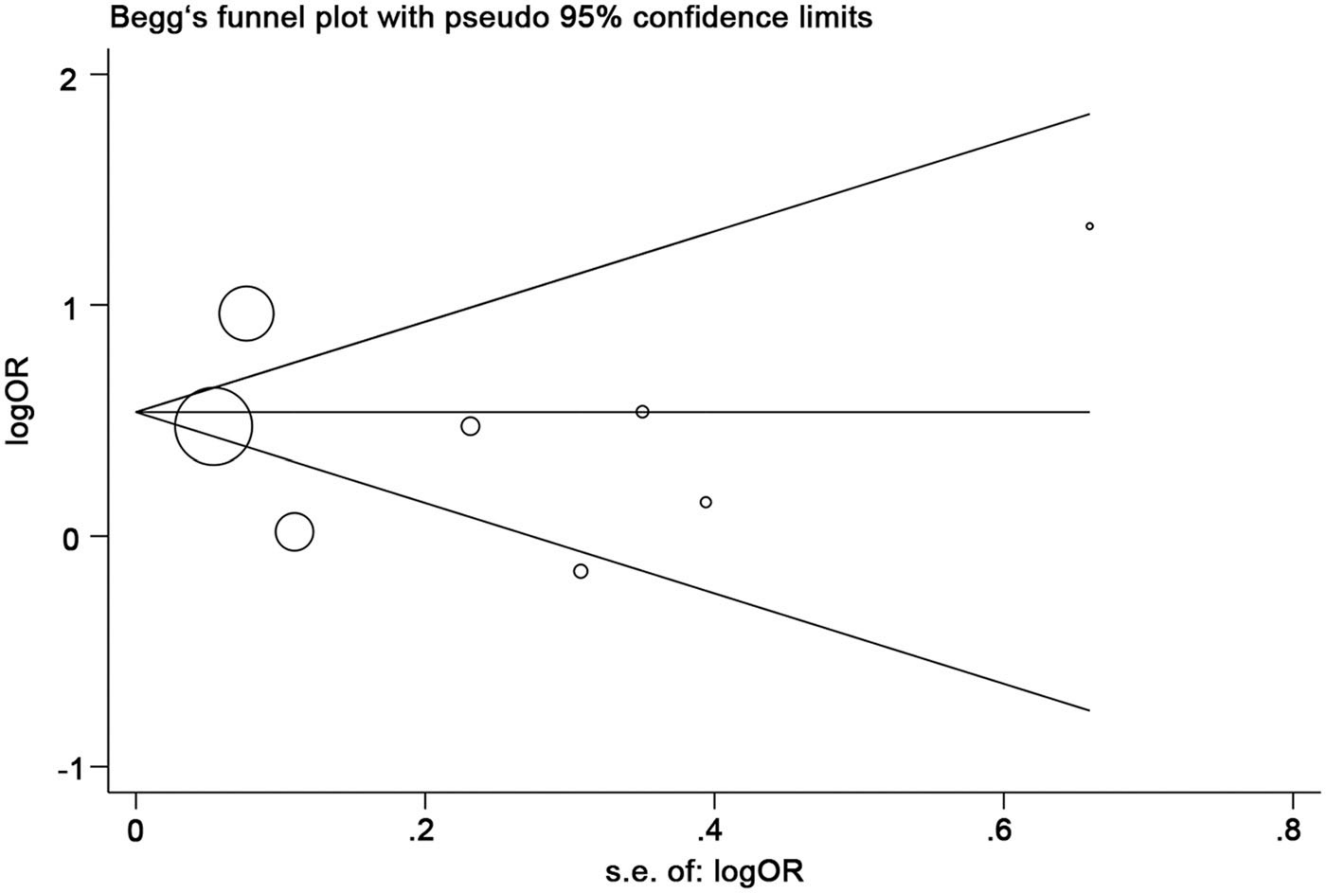
Begg’s funnel plot indicates no publication bias.

We conducted a meta-analysis to investigate the risk effect of vascular calcification on the prevalence of kidney stone. The heterogeneity (*I^2^* = 89%, *p* < 0.05) was substantial in our analysis, and random-effects model was used to evaluate the OR and its 95% CI. Pooled OR of kidney stone prevalence increased 1.54-fold in patients with vascular calcification compared with control participants (Figure 2: 95% CI: 1.13–2.10). Sensitivity analysis was conducted to illustrate the impact of each enrolled study. The consistent results after each study excluded indicated the robustness of our pooling analysis (Figure 3). There was no evidence of publication bias in the meta-analysis using Begg test, Egger test (Begg test = 0.457, Egger test = 0.675) and funnel plot (Figure 4).

**Figure 5.**
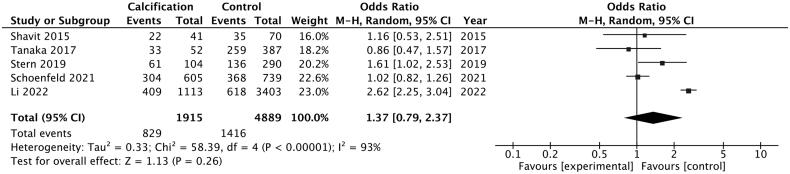
Pooled odds ratio of kidney stone disease in patients with abdominal aortic calcification compared with healthy control.

Since most studies, five out of seven, described patients with abdominal aortic calcification, we then conducted subgroup analysis trying to decipher the impact of abdominal calcification on kidney stone risk [25,26,33–35]. The pooled analysis showed a higher risk of kidney stone in patients with abdominal aortic calcification while the result was not significant (Figure 5: OR = 1.37, 95% CI: 0.79–2.37). No publication bias was noticed here (Begg test = 0.624, Egger test = 0.362).

**Figure 6.**
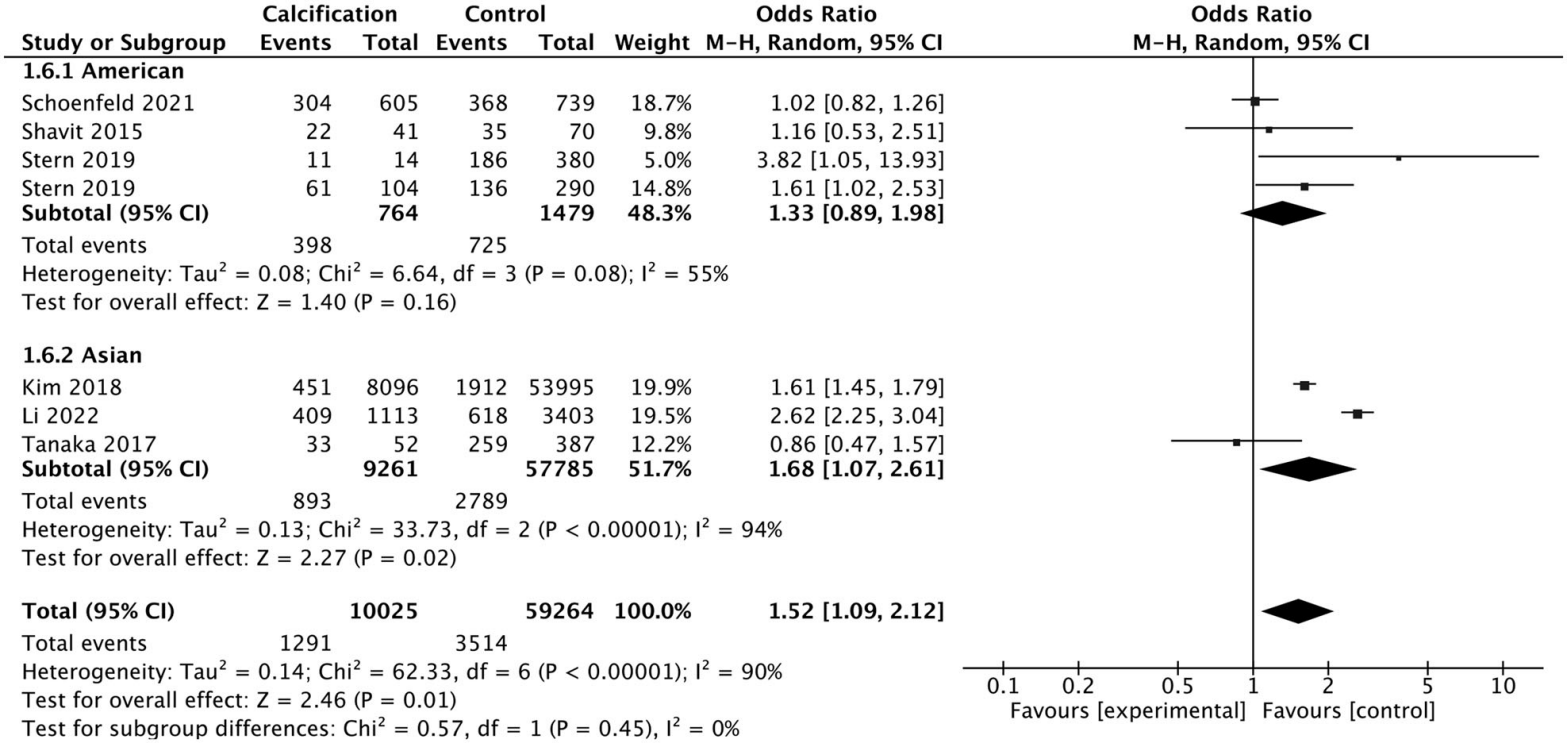
Pooled odds ratio of kidney stone disease in patients from different regions.

After the stratification of studies according to the regions, we found two studies conducted in America, one study conducted in the United Kingdom, three studies conducted in Asia and one in Arab state. Subgroup analysis according to the regions of participants indicated a significant higher risk of kidney stone risk in Asian participants (Figure 6: OR = 1.68, 95%CI: 1.07–2.61), which was not observed in American participants (OR = 1.33, 95%CI: 0.89-1.98). The heterogeneity was substantial here (*I^2^* = 55% for American subgroup, *I^2^* = 94% for Asian subgroup) and no publication bias was observed (Begg test = 0.174, Egger test = 0.180 for American subgroup, Begg test = 0.601, Egger test = 0.901 for Asian subgroup).

## Discussion

This meta-analysis provides the first comprehensive evidence for the association of vascular calcification with kidney stone risk. We demonstrated an overall 54% increased risk of kidney stone disease in patients with nonrenal vascular calcification in comparison with controls. This association persisted in sensitivity analysis. The enrolled studies reported vascular calcification in abdominal aorta, coronary, splenic and carotid artery. In single-study comparisons of populations with coronary or splenic artery calcification versus control, we observed an obvious increased risk of kidney stone in patients with vascular calcification. In pooled analysis of patients with abdominal aortic calcification, nonsignificantly increased risk of kidney stone disease was generated. Coronary artery calcification is a well-defined causal factor for cardiovascular events [36]. Abdominal aorta calcification is another independent predictor of cardiovascular events [37,38]. Previous meta-analysis and observational studies have pointed out the predicted higher risk of cardiovascular disease in kidney stone formers [9,39,40]. Given the epidemiological facts, we assumed the association between vascular calcification or their subtypes and kidney stone risk. However, among enrolled studies focusing on abdominal aorta calcification, Tanaka et al. included a small sample size with mere 52 patients diagnosed with vascular calcification. Moreover, renal insufficiency bias existed between kidney stone group and control [35]. Racial diversity marked another study focusing on abdominal aorta calcification [26]. Other unsolvable cofounding factors like age, gender and stone type may as well contributed to the negative result in abdominal aorta calcification subgroup analysis. Since abdominal CT or ultrasonography scans have become routine examinations in more health centers, abdominal aorta calcification is becoming more easily recognized [41]. Coronary or carotid artery scans are performed more often in clinical centers for screening risks of cardiovascular disease [31,42]. Based on our analysis results, patients recognized with vascular calcification should be treated more cautiously for their risk in kidney stone disease.

After we stratified studies according to regions, we found the higher risk of kidney stone disease was significant in Asian participants, but not significant in Americans. Nevertheless, the population enrolled in Asian studies reached 67,046, which is far larger than that in American studies with mere 2243 participants in total. Moreover, the composition of participants in American studies is more comlex, including Hispanics, non-Hispanic blacks and whites [26]. We believe the trend of higher risk of kidney stone disease in American participants with vascular calcification will be clarified when more participants get involved and confounding factors including demographic bias and clinical comorbidities get better stratification.

Calcium phosphate deposition, mainly in the form of hydroxyapatite, is the hallmark of vascular calcification, which also constitutes the second largest composition of kidney stones [43,44]. Oxalate burden, which contributes to calcium oxalate stone formation, is also regarded as an important role in vascular calcification [45]. The kidney stone plaque is similar with plaques formed during vascular calcification, both of which consisted of crystals mixed with organic matrix [46]. As more researches have pointed out the shared pathophysiologic pathways in forming calcium kidney stones and vascular calcification, the conception that both diseases are forms of pathological biomineralization or ectopic calcification is becoming generally accepted [47]. Despite the efforts in exploration of potent mechanisms in kidney stone and vascular calcification, there are still a lot remained revealing. Our work strengthened the connection behind the two diseaseas, and patients with vascular calcification are worthy of more caution for their risk of kidney stone and renal injury.

## Limitation

Our work has several limitations. The quantity of enrolled studies is small. Observational studies have inherent limitations, and we could not address direct interaction and draw causal conclusion. The majority of enrolled studies are case-control studies, which have adjusted for a wide variety of potential confounders like age, gender, smoking history and comorbidities. However, the residual confounding factors which contributed to both kidney stone and vascular calcification can still be substantial and cannot be excluded. Despite the majority of studies utilized X-ray photography or CT in diagnosing, Kim et al. and Li et al. also adopted ultrasound, which was not as effective [31,33]. In addition, we could not address the implication of stone types due to the limited information. Substantial heterogeneity was observed across enrolled studies with differences in vascular calcification types, populations, sample size, and diagnostic techniques. Therefore, additional large-scale investigations are necessary to validate the risk impact of vascular calcification on kidney stone.

## Conclusion

Patients with vascular calcification may be associated with an increased risk of kidney stone disease. The assumed predictive value of vascular calcification on kidney stone disease informed physicians, specifically nephrologists, of the potential impact on kidney injury with future risk for chronic kidney disease, subsequent cardiovascular morbidities and mortalities.

## Supplementary Material

Supplemental Material

Supplemental Material

## References

[1] Thongprayoon C, Krambeck AE, Rule AD. Determining the true burden of kidney stone disease. Nat Rev Nephrol. 2020;16:736–746. 32753740 10.1038/s41581-020-0320-7

[2] Liu Y, Chen Y, Liao B, et al. Epidemiology of urolithiasis in Asia. Asian J Urol. 2018;5:205–214. 30364478 10.1016/j.ajur.2018.08.007 PMC6197415

[3] Sorokin I, Mamoulakis C, Miyazawa K, et al. Epidemiology of stone disease across the world. World J Urol. 2017;35(9):1301–1320. 28213860 10.1007/s00345-017-2008-6

[4] Xu LHR, Adams-Huet B, Poindexter JR, et al. Temporal changes in kidney stone composition and in risk factors predisposing to stone formation. J Urol. 2017;197(6):1465–1471. 28111301 10.1016/j.juro.2017.01.057 PMC5433898

[5] Yang C, Wang H, Zhao X, et al. CKD in China: evolving spectrum and public health implications. Am J Kidney Dis. 2020;76(2):258–264. 31492486 10.1053/j.ajkd.2019.05.032

[6] Alexander RT, Hemmelgarn BR, Wiebe N, et al. Kidney stones and kidney function loss: a cohort study. BMJ. 2012;345:e5287. 22936784 10.1136/bmj.e5287 PMC3431443

[7] Dhondup T, Kittanamongkolchai W, Vaughan LE, et al. Risk of ESRD and mortality in kidney and bladder stone formers. Am J Kidney Dis. 2018;72(6):790–797. 30146423 10.1053/j.ajkd.2018.06.012 PMC6252145

[8] Webster AC, Nagler EV, Morton RL, et al. Chronic kidney disease. Lancet. 2017;389(10075):1238–1252. 27887750 10.1016/S0140-6736(16)32064-5

[9] Rule AD, Roger VL, Melton LJ, 3rd, et al. Kidney stones associate with increased risk for myocardial infarction. J Am Soc Nephrol. 2010;21(10):1641–1644. 20616170 10.1681/ASN.2010030253 PMC3013539

[10] Ferraro PM, Taylor EN, Eisner BH, et al. History of kidney stones and the risk of coronary heart disease. JAMA. 2013;310(4):408–415. 23917291 10.1001/jama.2013.8780 PMC4019927

[11] Kim SY, Bang WJ, Min C, et al. Association of nephrolithiasis with the risk of cardiovascular diseases: a longitudinal follow-up study using a national health screening cohort. BMJ Open. 2020;10(11):e040034. 10.1136/bmjopen-2020-040034 PMC7668357 33191264

[12] Razavi AC, Agatston AS, Shaw LJ, et al. Evolving role of calcium density in coronary artery calcium scoring and atherosclerotic cardiovascular disease risk. JACC Cardiovasc Imaging. 2022;15(9):1648–1662. 35861969 10.1016/j.jcmg.2022.02.026 PMC9908416

[13] Anugula D, Cardoso R, Grandhi GR, et al. Extra-coronary calcification and cardiovascular events: what do we know and where are we heading? Curr Atheroscler Rep. 2022;24(10):755–766. 36040566 10.1007/s11883-022-01051-5

[14] O'Connor SD, Graffy PM, Zea R, et al. Does nonenhanced CT-based quantification of abdominal aortic calcification outperform the Framingham risk score in predicting cardiovascular events in asymptomatic adults? Radiology. 2019;290(1):108–115. 30277443 10.1148/radiol.2018180562

[15] Lanzer P, Hannan FM, Lanzer JD, et al. Medial arterial calcification: JACC state-of-the-Art review. J Am Coll Cardiol. 2021;78:1145–1165. 34503684 10.1016/j.jacc.2021.06.049 PMC8439554

[16] Coe FL, Parks JH, Asplin JR. The pathogenesis and treatment of kidney stones. N Engl J Med. 1992;327(16):1141–1152. 1528210 10.1056/NEJM199210153271607

[17] Carbone A, Al Salhi Y, Tasca A, et al. Obesity and kidney stone disease: a systematic review. Minerva Urol Nefrol. 2018;70(4):393–400. 29856171 10.23736/S0393-2249.18.03113-2

[18] Witting C, Langman CB, Assimos D, et al. Pathophysiology and treatment of enteric hyperoxaluria. Clin J Am Soc Nephrol. 2021;16:487–495. 32900691 10.2215/CJN.08000520 PMC8011014

[19] Liu CJ, Cheng CW, Tsai YS, et al. Crosstalk between renal and vascular calcium signaling: the link between nephrolithiasis and vascular calcification. Int J Mol Sci. 2021;22(7):3590. 33808324 10.3390/ijms22073590 PMC8036726

[20] Yiu AJ, Callaghan D, Sultana R, et al. Vascular calcification and stone disease: a new look towards the mechanism. J Cardiovasc Dev Dis. 2015;2(3):141–164. 26185749 10.3390/jcdd2030141 PMC4501032

[21] Letavernier E, Bouderlique E, Zaworski J, et al. Pseudoxanthoma elasticum, kidney stones and pyrophosphate: from a rare disease to urolithiasis and vascular calcifications. Int J Mol Sci. 2019;20(24):6353. 31861118 10.3390/ijms20246353 PMC6940945

[22] Saenz-Medina J, Munoz M, Rodriguez C, et al. Endothelial dysfunction: an intermediate clinical feature between urolithiasis and cardiovascular diseases. Int J Mol Sci. 2022;23(2):912. 35055099 10.3390/ijms23020912 PMC8778796

[23] Reiner AP, Kahn A, Eisner BH, et al. Kidney stones and subclinical atherosclerosis in young adults: the CARDIA study. J Urol. 2011;185(3):920–925. 21251678 10.1016/j.juro.2010.10.086 PMC3827917

[24] Hsi RS, Spieker AJ, Stoller ML, et al. Coronary artery calcium score and association with recurrent nephrolithiasis: the Multi-Ethnic study of atherosclerosis. J Urol. 2016;195(4 Pt 1):971–976. 26454103 10.1016/j.juro.2015.10.001 PMC4966606

[25] Stern KL, Ward RD, Li J, et al. Nonrenal systemic arterial calcification predicts the formation of kidney stones. J Endourol. 2019;33(12):1032–1034. 31220925 10.1089/end.2019.0243

[26] Schoenfeld D, Zhu D, Mohn L, et al. The relationship between vascular calcifications and urolithiasis in a large, multiethnic patient population. Urolithiasis. 2021;49(6):533–541. 33961080 10.1007/s00240-021-01268-0

[27] Liberati A, Altman DG, Tetzlaff J, et al. The PRISMA statement for reporting systematic reviews and meta-analyses of studies that evaluate healthcare interventions: explanation and elaboration. BMJ. 2009;339:b2700. 19622552 10.1136/bmj.b2700 PMC2714672

[28] Lo CK, Mertz D, Loeb M. Newcastle-Ottawa scale: comparing reviewers’ to authors’ assessments. BMC Med Res Methodol. 2014;14:45. 24690082 10.1186/1471-2288-14-45 PMC4021422

[29] Stang A. Critical evaluation of the Newcastle-Ottawa scale for the assessment of the quality of nonrandomized studies in meta-analyses. Eur J Epidemiol. 2010;25(9):603–605. 20652370 10.1007/s10654-010-9491-z

[30] Lin L, Chu H. Quantifying publication bias in meta-analysis. Biometrics. 2018;74(3):785–794. 29141096 10.1111/biom.12817 PMC5953768

[31] Kim S, Chang Y, Sung E, et al. Association between sonographically diagnosed nephrolithiasis and subclinical coronary artery calcification in adults. Am J Kidney Dis. 2018;71(1):35–41. 28823586 10.1053/j.ajkd.2017.06.026

[32] Patil S, Maheshwari S, Khandelwal S, et al. Prevalence of calcified carotid artery atheromas on panoramic radiographs of renal stone patients. Saudi J Kidney Dis Transpl. 2016;27(1):62–66. 10.4103/1319-2442.174074 26787568

[33] Li B, Tang Y, Zhou L, et al. Association between aortic calcification and the presence of kidney stones: calcium oxalate calculi in focus. Int Urol Nephrol. 2022;54(8):1915–1923. 34846621 10.1007/s11255-021-03058-4 PMC9262773

[34] Shavit L, Girfoglio D, Vijay V, et al. Vascular calcification and bone mineral density in recurrent kidney stone formers. Clin J Am Soc Nephrol. 2015;10(2):278–285. 25635036 10.2215/CJN.06030614 PMC4317743

[35] Tanaka T, Hatakeyama S, Yamamoto H, et al. Clinical relevance of aortic calcification in urolithiasis patients. BMC Urol. 2017;17(1):25. 28376750 10.1186/s12894-017-0218-2 PMC5379761

[36] Hermann DM, Gronewold J, Lehmann N, Heinz Nixdorf Recall Study Investigative Group, et al. Coronary artery calcification is an independent stroke predictor in the general population. Stroke. 2013;44(4):1008–1013. 23449263 10.1161/STROKEAHA.111.678078

[37] Davila JA, Johnson CD, Behrenbeck TR, et al. Assessment of cardiovascular risk status at CT colonography. Radiology. 2006;240(1):110–115. 16793974 10.1148/radiol.2401050948

[38] Bastos Goncalves F, Voute MT, Hoeks SE, et al. Calcification of the abdominal aorta as an independent predictor of cardiovascular events: a meta-analysis. Heart. 2012;98(13):988–994. 22668866 10.1136/heartjnl-2011-301464

[39] Liu Y, Li S, Zeng Z, et al. Kidney stones and cardiovascular risk: a meta-analysis of cohort studies. Am J Kidney Dis. 2014;64(3):402–410. 24797522 10.1053/j.ajkd.2014.03.017

[40] Domingos F, Serra A. Nephrolithiasis is associated with an increased prevalence of cardiovascular disease. Nephrol Dial Transplant. 2011;26(3):864–868. 20709737 10.1093/ndt/gfq501

[41] Amin A, Rejjal A, McDonald P, et al. Nephrocalcinosis, cholelithiasis, and umbilical vein calcification in a premature-infant. Abdom Imaging. 1994;19(6):559–560. 7820035 10.1007/BF00198265

[42] Bos D, Arshi B, van den Bouwhuijsen QJA, et al. Atherosclerotic carotid plaque composition and incident stroke and coronary events. J Am Coll Cardiol. 2021;77(11):1426–1435. 33736825 10.1016/j.jacc.2021.01.038

[43] Villa-Bellosta R. Vascular calcification: key roles of phosphate and pyrophosphate. Int J Mol Sci. 2021;22(24):13536. 34948333 10.3390/ijms222413536 PMC8708352

[44] Lieske JC, Rule AD, Krambeck AE, et al. Stone composition as a function of age and sex. Clin J Am Soc Nephrol. 2014;9(12):2141–2146. 25278549 10.2215/CJN.05660614 PMC4255407

[45] Mulay SR, Eberhard JN, Pfann V, et al. Oxalate-induced chronic kidney disease with its uremic and cardiovascular complications in C57BL/6 mice. Am J Physiol Renal Physiol. 2016;310(8):F785–F795. 26764204 10.1152/ajprenal.00488.2015 PMC5504458

[46] Khan SR, Canales BK, Dominguez-Gutierrez PR. Randall’s plaque and calcium oxalate stone formation: role for immunity and inflammation. Nat Rev Nephrol. 2021;17(6):417–433. 33514941 10.1038/s41581-020-00392-1

[47] Khan SR. Reactive oxygen species, inflammation and calcium oxalate nephrolithiasis. Transl Androl Urol. 2014;3:256–276. 25383321 10.3978/j.issn.2223-4683.2014.06.04 PMC4220551

