# Supplementary material for: Vascular calcification on the risk of kidney stone: a meta-analysis
Source: Ren Fail. 2023 Mar 3;45(1):2183727. doi: 10.1080/0886022X.2023.2183727 (PMC9987734; doi:10.1080/0886022X.2023.2183727)
Supplement: Supplemental Material [file IRNF_A_2183727_SM8603.pdf]

Supplementary table 2. Search strategy in PubMed

Search	Query	Items found
#1	((((((((((((kidney calculi[MeSH Terms]) OR (urolithiasis[MeSH Terms])) OR (nephrolithiasis[MeSH Terms])) OR (calculus, kidney)) OR (kidney calculus)) OR (nephrolith)) OR (renal calculus)) OR (kidney stones)) OR (kidney stone)) OR (stone, kidney)) OR (stones, kidney)) OR (renal calculi)) OR (calculi, renal) OR (calculus, renal)	49,956
#2	(((((((vascular calcification[MeSH Terms]) OR (calcification, vascular)) OR (calcifications, vascular)) OR (vascular calcifications)) OR (vascular calcinosis)) OR (calcinoses, vascular)) OR (calcinosis, vascular)) OR (vascular calcinoses)	21,174
#3	#1 AND #2	146

Supplementary table 2. Search strategy in Embase

Search	Query	Items found
#1	'nephrolithiasis'/exp OR 'nephrolithiasis'	36,059
#2	'kidney calcification'/exp OR 'kidney calcification'	6,472
#3	'kidney calyx'/exp OR 'kidney calyx'	2,507
#4	'blood vessel calcification'/exp OR 'blood vessel calcification'	26,875
#5	'artery calcification'/exp OR 'artery calcification'	20,809
#6	#1 OR #2 OR #3	42,938
#7	#4 OR #5	27,967
#8	#6 AND #7	278

Information Classification: General

Supplementary table 2. Search strategy in Web of Science

Search	Query	Items found
#1	(((((((TS=(Vascular Calcification)) OR TS=(Calcification, Vascular)) OR TS=(Calcifications, Vascular)) OR TS=(Vascular Calcifications)) OR TS=(Vascular Calcinosis)) OR TS=(Calcinoses, Vascular)) OR TS=(Calcinosis, Vascular)) OR TS=(Vascular Calcinoses)	13,854
#2	((((((((((((((((TS=(Kidney Calculi)) OR TS=(Urolithiasis)) OR TS=(Nephrolithiasis)) OR TS=(Calculi, Kidney)) OR TS=(Calculus, Kidney)) OR TS=(Kidney Calculus)) OR TS=(Nephrolith)) OR TS=(Renal Calculus)) OR TS=(Kidney Stones)) OR TS=(Kidney Stone)) OR TS=(Stone, Kidney)) OR TS=(Stones, Kidney)) OR TS=(Renal Calculi)) OR TS=(Calculi, Renal)) OR TS=(Calculus, Renal)) OR TS=(Urinary Lithiasis)) OR TS=(Lithiasis, Urinary)	30,671
#3	#1 AND #2	115

Supplementary table 2. Search strategy in Cochrane Library

Search	Query	Items found
#1	Nephrolithiasis	1,747
#2	Urolithiasis	915
#3	Kidney Calculi	1,282
#4	Vascular Calcification	824
#5	Calcinosis	464
#6	Blood Vessels	6,612

Information Classification: General

#7	Arteries	9,736
#8	#6 OR #7	15,261
#9	#8 AND 5	9,149
#10	#4 AND #9	106
#11	#4 OR #10	824
#12	#1 OR #2 OR #3	3,064
#13	#11 AND #12	8

Information Classification: General
